# An exact analysis of the temperature control of optical waveguides

**DOI:** 10.1007/s11082-025-08076-5

**Published:** 2025-03-11

**Authors:** Frank Payne, Zipei Song, Mohan Wang, Julian A. J. Fells

**Affiliations:** 1 Lincoln College, University of Oxford, Turl Street, Oxford, OX1 3DR UK; 2 Department of Engineering Science, University of Oxford, Parks Road, Oxford, OX1 3PJ UK

**Keywords:** Athermal waveguide, Temperature compensated waveguide, Optical fiber, Fiber Bragg grating, Arrayed waveguide grating, Micro-channel fiber

## Abstract

In this paper we present an exact analysis of the variation with temperature of the effective index of an arbitrary optical waveguide. Our results allow the design of temperature compensated devices using only a single parameter calculated at one temperature avoiding the need to perform an analysis over a range of temperatures. We derive expressions for both weakly and strongly guiding waveguides. We derive a complete analytical solution for the design of micro channel fibers where the micro channels are filled with a temperature compensating material whose refractive index equals that of the fiber cladding at a specified reference temperature. We also analyse the more general case including the effects of thermal expansion. We illustrate our analysis with the application to athermal fiber Bragg gratings and arrayed waveguide grating filters.

## Introduction

The propagation of light through an optical waveguide is affected by changes of temperature in two important ways: (i) thermal changes in the refractive indices of the waveguide materials, and (ii) thermal expansion of the waveguide or the substrate on which the waveguide is grown. The former results in a change with temperature of the effective index $n_{\text {eff}}$ of the waveguide mode, and the latter causes a change of phase along the waveguide because of changes of the waveguide dimensions. Both effects usually occur together and have an impact on the performance of a wide range of waveguide devices. For example, fiber Bragg gratings find many applications as sensors and optical filters (Kersey et al. 1997), and arrayed waveguide gratings (AWGs) have applications as wavelength division multiplexers and demultiplexers, as well as wavelength routers (Leijtens et al. 2006). In the case of a fiber Bragg grating, a change in temperature will cause a shift in the Bragg wavelength and impair the sensor response. In the case of an AWG, a change in temperature will cause a shift in the filter’s center wavelength and free spectral range, as well as misalignment with the ITU grid (Leijtens et al. 2006; Okamoto 2000). Other examples include systems where variations with temperature of the propagation time through an optical fiber adversely affects performance. Examples include the synchronization of optical data signals and clocks. A review of these and other applications can be found in Fokoua et al. (2017).

Most waveguide cores are made from materials with a positive thermo-optic coefficient *dn*/*dT*, and a common way to mitigate the effects of temperature change is by incorporating materials with a negative thermo-optic coefficient into the waveguide design. Athermal AWGs made using silica-on-silicon technology (Himeno et al. 1998; Ou 2003) have been demonstrated. In Inoue et al. (1997); Kamei et al. (2009) a silicone wedge is inserted into a groove cut into the array of silica waveguides, and in Li et al. (2007) a polymer cladding with negative *dn*/*dT* is used. A similar approach has been applied to AWGs made with silicon-on-insulator technology (Jalali et al. 1998; Ye et al. 2008; Wang et al. 2009, 2012), and all-polymer AWGs (Keil et al. 2001).

The athermal design of fiber Bragg gratings has been demonstrated by creating microchannels around the fiber core which are filled with a liquid with a negative thermo-optic coefficient (Man et al. 2019; Mothe et al. 2008; Huy et al. 2008; Song et al. 2025).

The published literature describes a variety of methods for reducing the effects of temperature on waveguide devices. A common feature is a theoretical model for the variation of the modal effective index with temperature. Several different models have been reported. In some cases the temperature variation of $n_{\text {eff}}$ is related to the refractive indices of the waveguide core and cladding, weighted in proportion to the modal power carried by each. In others, $dn_{\text {eff}}/dT$ is related to the rate of change of core and cladding indices, also weighted by the modal power fraction carried by each. There is no detailed derivation of any of these approximate results. It is the purpose of this paper to present an exact and rigorous analysis of the variation of $n_{\text {eff}}$ with temperature, and to derive exact equations for $dn_{\text {eff}}/dT$ that allows the design of a wide range of waveguide devices without the need for device modelling over a range of temperatures. Our analysis is valid for weakly guiding waveguides where the modes are described by the scalar wave equation. This is not a strong restriction as many waveguides are weakly guiding. We also derive the corresponding result for waveguides with large refractive index differences and which must be described by full vector modes, including birefringent waveguides.

In this paper we distinguish between temperature compensated waveguides and athermal waveguides. The former refers to waveguides in which only the temperature variation of the effective index is minimized, and the latter refers to waveguide devices where the effects of both thermal expansion and changes in the effective index are compensated.

This paper has eight sections. In Sect. 2 we discuss the temperature dependence of a step index optical fiber for which a direct calculation of $dn_{\text {eff}}/dT$ is possible from first principles. We show how this result can be used to design and model a temperature compensated fiber. Section 3 derives an exact expression for $dn_{\text {eff}}/dT$ valid for an arbitrary weakly guiding waveguide structure and which relates $dn_{\text {eff}}/dT$ to the modal power fractions in each waveguide region. The case where the temperature compensating regions have a refractive index equal to that of the fiber cladding at a particular reference temperature is analysed in Sect. 4. We consider fibers with microchannels consisting of either an annulus sector shape or side hole shape. For these cases the modal overlap fractions can be calculated exactly and it is possible to produce exact analytical results for temperature compensated designs with minimal computation. In Sect. 5 we discuss the more general situation where the refractive index of the compensating regions differs from that of the cladding. In Sects. 2, 4, and 5 we discuss practical ways of fabricating the waveguides analyzed, and we give estimates of the waveguide losses.

An athermal design must include the effects of thermal expansion, and we discuss this in detail in Sect. 6. For waveguides with large refractive index differences the weakly guiding scalar analysis is not valid and a full vector treatment is necessary. This is set out in detail in Sect. 7.

In Sect. 8 we compare our approach, which requires the calculation of only one paramater at one temperature, to that of others where the waveguide response must be computed over a range of temperatures. Finally, in Sect. 8 we present our conclusions.

## Thermal analysis of the step index optical fiber

It is instructive to consider the temperature dependence of a step index optical fiber. The step index fiber is one of only a few examples where a direct calculation is possible of the temperature variation $dn_{\text {eff}}/dT$ of the guided mode effective index.

We consider a weakly guiding optical fiber with core radius *a*, and refractive index $n_{1}$, surrounded by an infinite cladding of refractive index $n_{2}$. The fundamental $\text {LP}_{01}$ mode is described by the scalar wave equation. The mode parameters *u*, *w* and *V* are defined by Okamoto (2000); Snyder and Love (1984); Adams (1981):

1 $$\begin{aligned} u=a\sqrt{n_{1}^{2}k^{2}-\beta ^{2}}~;~ w=a\sqrt{\beta ^{2}-n_{2}^{2}k^{2}} \end{aligned}$$

2 $$\begin{aligned} V = ak\sqrt{ n_{1}^{2}-n_{2}^{2}} = \sqrt{u^{2} + w^{2}} \end{aligned}$$

The propagation constant is $\beta$ and *k* is the free space wave number. The $\text {LP}_{01}$ modal field is given by:

3 $$\begin{aligned} {\psi } = {\left\{ \begin{array}{ll} AJ_{0}\left( \dfrac{ur}{a}\right) & { \text {for}}\ r \le a ,\\ {\hspace{4.25pt}} A\dfrac{J_{0}(u)}{K_{0}(w)}\cdot K_0{\left( \dfrac{wr}{a}\right) } & {\text {for}}\ r>a \end{array}\right. } \end{aligned}$$

where *J* is a Bessel function of the first kind, and *K* is a modified Bessel function of the first kind (Abramowitz and Stegun 2013; Gradshteĭn and Ryzhik 1980). The modal field is normalised so that:

4 $$\begin{aligned} \int _{0}^{\infty }\int _{0}^{2\pi }\psi ^{2}\,rdr\,d\theta = 1 \end{aligned}$$

The normalisation constant *A* is given by:

5 $$\begin{aligned} A = \frac{w}{aVJ_1(u)\sqrt{\pi }} \end{aligned}$$

In deriving Eq. (5) we have made use of the eigenvalue equation that determines the propagation constant:

6 $$\begin{aligned} \frac{J_{0}\left( u\right) }{uJ_{1}\left( u\right) } = \frac{K_{0}\left( w\right) }{wK_{1}\left( w\right) } \end{aligned}$$

Details of the derivation of Eqs. (1) to (6) can be found in Okamoto (2000); Snyder and Love (1984); Adams (1981). It is straightforward to show that the fractions of modal power in the core and cladding are given by:

7 $$\begin{aligned} \Gamma _{1} = \int _{0}^{a}\int _{0}^{2\pi }\psi ^{2}\,rdr\,d\theta = 1-\frac{u^{2}}{V^{2}}\cdot \left( 1-\frac{K_{0}^{2}(w)}{K_{1}^{2}(w)} \right) \end{aligned}$$

8 $$\begin{aligned} \Gamma _{2} = \int _{a}^{\infty }\int _{0}^{2\pi }\psi ^{2}\,rdr\,d\theta = \frac{u^{2}}{V^{2}}\cdot \left( 1-\frac{K_{0}^{2}(w)}{K_{1}^{2}(w)} \right) \end{aligned}$$

The effective index $n_{\text {eff}}$ is defined by $n_{\text {eff}}=\beta /k$. From Eq. (6), *u* is a function only of *V*, and from Eqs. (1) and (2) the effective index can be written as:

9 $$\begin{aligned} n_{\text {eff}}^2 = n_{1}^{2} - (n_{1}^{2}-n_{2}^{2})f(V) \end{aligned}$$

where $f(V)=u^{2}/V^{2}$. Differentiating Eq. (9) we obtain:

10 $$\begin{aligned} \frac{dn_{\text {eff}}^{2}}{dT}=\frac{dn_{1}^{2}}{dT}(1-f(V)) +\frac{dn_{2}^{2}}{dT}f(V) - (n_{1}^{2}-n_{2}^{2})\frac{df}{dV}\cdot \frac{dV}{dT} \end{aligned}$$

From the definition of *V* in Eq. (2) we find *dV*/*dT* is given by:

11 $$\begin{aligned} \frac{dV}{dT} = \frac{V}{2(n_{1}^{2}-n_{2}^{2})}\cdot \left( \frac{dn_{1}^{2}}{dT} - \frac{dn_{2}^{2}}{dT} \right) \end{aligned}$$

and *df*/*dV* is given by:

12 $$\begin{aligned} \frac{df}{dV}=\frac{2u}{V^2}\left( \frac{du}{dV}-\frac{u}{V}\right) \end{aligned}$$

Equation (12) includes *du*/*dV* which can be shown to be (Okamoto 2000):

13 $$\begin{aligned} \frac{du}{dV}=\frac{u}{V}\cdot \left[ 1-\frac{K_0^2(w)}{K_1^2(w)}\right] \end{aligned}$$

Combining Eqs. (10) to (13) gives the following result:

14 $$\begin{aligned} \frac{dn_{\text {eff}}^2}{dT}=\frac{dn_1^2}{dT}\cdot \left[ 1-\frac{u^2}{V^2}\left( 1-\frac{K_0^2\left( w\right) }{K_1^2\left( w\right) }\right) \right] +\frac{dn_2^2}{dT}\cdot \left[ \frac{u^2}{V^2}\left( 1-\frac{K_0^2\left( w\right) }{K_1^2\left( w\right) }\right) \right] \end{aligned}$$

**Fig. 1:**
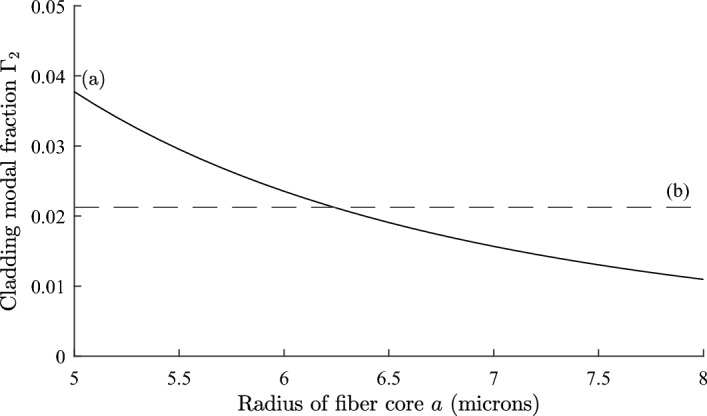
Plot of $\Gamma _{2}$, fraction of power in cladding as a function of core radius *a* for a step index fiber with cladding refractive index 1.434 curve** a**. The horizontal line** d** indicates where $\Gamma _{2} = 0.0212$

The effects of transverse and longitudinal thermal expansion have not been included in the derivation of Eq. (14). We consider these in Section 6. From Eqs. (7) and (8) we recognise the expressions in square brackets on the right hand side of Eq. (14) as $\Gamma _{1}$ and $\Gamma _{2}$, the fractions of modal power in the core and cladding, so that Eq. (14) can be written more simply as:

15 $$\begin{aligned} \frac{dn_{\text {eff}}^2}{dT} = \Gamma _{1}\frac{dn_1^2}{dT} + \Gamma _{2}\frac{dn_2^2}{dT} \end{aligned}$$

Equation (15) shows that the temperature dependence of $n^{2}_{\text {eff}}$ is given by the temperature dependence of $n^{2}_{1}$ and $n^{2}_{2}$ weighted by the fractions of modal power they each carry. Since $\Gamma _{1}+\Gamma _{2}=1$ we can rewrite Eq. (15) in the following form:

16 $$\begin{aligned} \frac{dn_{\text {eff}}^2}{dT}= \left( {1-\Gamma }_2\right) \frac{dn_1^2}{dT}+\Gamma _2\frac{dn_2^2}{dT} \end{aligned}$$

From Eq. (16) the condition for $dn_{\text {neff}}/dT=0$ is given by:

17 $$\begin{aligned} \Gamma _2=\dfrac{n_1\dfrac{dn_1}{dT}}{n_1\dfrac{dn_1}{dT}-n_2\dfrac{dn_2}{dT}} \end{aligned}$$

Equation (17) allows us to calculate the fraction of power in the fiber cladding that minimises the temperature variation of the waveguide mode’s effective index. We discuss this in more detail in the next section.

### Design of a temperature compensated step index fiber

We illustrate the theory developed in Sect. 2 with the design of a temperature compensated step index fibre which has $dn_{\text {eff}}/dT = 0$ at a reference temperature $T_{0}$. We assume a core refractive index $n_{1} = 1.451$, and $dn_{1}/dT = 8.45\times 10^{-6}/^{\circ }\text {C}$ at $T_0 = 25^{\circ } \text {C}$, which corresponds to fused silica (Leviton and Frey 2006). We examine the design for a cladding refractive index $n_{2} =$1.434 with a temperature dependence $dn_{2}/dT= -394\times 10^{-6}/^{\circ }\text {C}$, which is available from the range of siloxane and aliphatic / alicyclic hydrocarbon based refractive index liquids manufactured by Cargille Laboratories, data sheet: Refractive-Index-Liquid-Series-AA-n$-$1.4440-at$-$589.3-nm-and-$25^{\circ } \text {C}$ (Cargille). A wavelength of 1550 nm is assumed. From Eq. (17) the required value of $\Gamma _{2}$ is 0.0212. The modal power fraction $\Gamma _{2}$ was calculated as a function of the core radius *a*, using Eqs. (6) and (8). The results are shown in Fig. 1. The dashed line (b) drawn at $\Gamma _{2} = 0.0212$ shows the core radius for which a design can exist. From Fig. 1 the required core radius for a temperature compensated design is 6.24 µm.

**Fig. 2:**
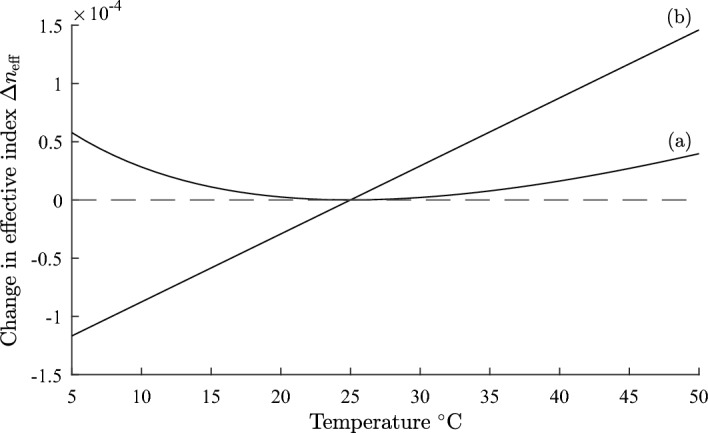
Plot of fractional change in effective index $\Delta n_{\text {eff}}$ against temperature for a step index fibre. Curve** a** is for a temperature compensated design with parameters defined in Sect. 2.1, and curve** b** is for an uncompensated fiber where core and cladding both vary as $dn/dT = 8.45\times 10^{-6}/^{\circ }\text {C}$

We define the fractional change in the effective index $\Delta n_{\text {eff}}$ by:

18 $$\begin{aligned} \Delta n_{\text {eff}} = \frac{n_{\text {eff}}(T)- n_{\text {eff}}(T_{0})}{n_{\text {eff}}(T_{0})} \end{aligned}$$

In Fig. 2, curve (a), $\Delta n_{\text {eff}}$ is shown as a function of temperature *T* for a step index fiber with a core radius of 6.24 µm and with the other parameters given above. Also, shown for comparison in curve (b), is the variation of $\Delta n_{\text {eff}}$ with temperature for an uncompensated fiber where both core and cladding indices vary as $dn/dT = 8.45\times 10^{-6}/^{\circ }\text {C}$. For the temperature compensated design the maximum variation of $\Delta n_{\text {eff}}$ is about $5\times 10^{-5}$, whereas for the uncompensated fiber it is $26.4\times 10^{-5}$. The predicted zero of $dn_\text {eff}/dT$ at $25^{\circ } \text {C}$ is evident in Fig. 2. Using Eq. (17) the single plot of $\Gamma _{2}$ in Fig. 1 gives the value of the fiber core for which a temperature compensated design can exist for the parameters specified and without the need to perform repeated calculations of $\Delta n_{\text {eff}}$ as a function of temperature.

The simplicity of the step index fiber design raises the question of practical methods of fabrication. There are two widely used techniques described in the literature. The first is based on chemical etching (Ascorbe et al. 2016; Meunier et al. 2023; Zaca-Morán et al. 2018; Kim et al. 2012). In this method the outer protective acrylate coating of the fiber is removed over a length from a few mm to a few cms. The outer cladding of the fiber is then gradually removed by etching in a solution of hydrofluoric acid (HF). By varying the concentration of HF, and the etching time, it is possible to completely remove the fiber cladding and expose the fiber core which can then be immersed in the temperature controlling refractive index liquid. This method has been used to demonstrate a temperature compensated fiber Bragg grating (Kim et al. 2012).

The second method of fabrication is based on tapering the fiber by the heat and draw method (Hale et al. 1996; Hale and Payne 1994; Pendock et al. 1993; Henry and Payne 1995; Gao et al. 2012). In this method the outer protective acrylate fiber coating is removed and a section of bare fiber is heated in an oxy-butane or oxy-propane flame. As the fiber melts it is extended so that the original cladding is reduced to a few microns and the original core is reduced to fractions of a micron. By careful control an adiabatic taper results so that the waveguide mode gradually evolves from one guided by the original fiber core to one guided by the reduced diameter cladding which allows interaction with the surrounding temperature controlling liquid. A temperature compensated fiber Bragg grating has also been demonstrated using this method (Gao et al. 2012).

The optical loss of the temperature compensated step index fiber can be estimated from the loss of the refractive index liquid. For the Cargille Laboratories liquid cited above the optical transmission is quoted as $76\%$ over one cm, corresponding to an optical loss of $L=1.2 \text {dB/cm}$ (Cargille). The fraction of optical power propagating in the liquid is $\Gamma _{2}$ so that the combined loss is given by $\Gamma _{2}L$ (Adams 1981). For $\Gamma _{2}=0.0212$ this corresponds to a device loss of $0.025\text {dB/cm}$.

## Thermal analysis of a general optical waveguide

**Fig. 3:**
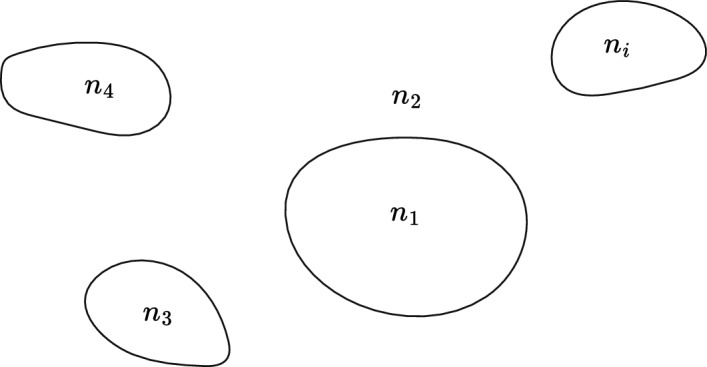
A general optical waveguide with core of refractive index $n_{1}$, infinite cladding of index $n_{2}$ and temperature controlling regions of index $n_{3}, n_{4}, \cdots , n_{i}$

In this section we derive an exact expression for the rate of change with temperature of the effective index of a weakly guiding optical waveguide shown in cross section in Fig. 3.

The core is of arbitrary cross section with refractive index $n_{1}$ and the infinite cladding has index $n_{2}$. Within the cladding are temperature controlling regions of arbitrary shape, parallel to the waveguide axis. For completeness, we consider the general case where the refractive indices $n_{3}, n_{4},\cdots n_{i}$ can take arbitrary values. In the weakly guiding approximation, and at temperature *T*, the modal wave function $\psi =\psi (T)$ satisfies the scalar wave equation (Snyder and Love 1984):

19 $$\begin{aligned} \nabla _{t} ^{2}\psi +k^{2}n^{2}\psi = \beta ^{2}\psi \end{aligned}$$

where

20 $$\begin{aligned} \nabla _{t}^{2}=\frac{\partial }{\partial x^{2}} + \frac{\partial }{\partial y^{2}} \end{aligned}$$

The refractive index varies with temperature as $n=n(T)$, the free space wave number is *k*, and the propagation constant is $\beta =\beta (T)$. At temperature $\overline{T}=T+\delta T$ the wave equation becomes

21 $$\begin{aligned} \nabla _{t} ^{2}\overline{\psi }+k^{2}\overline{n}^{2}\overline{\psi } = \overline{\beta }^{2}\overline{\psi } \end{aligned}$$

where $\overline{\psi }=\psi (\overline{T})$, $\overline{n}=n(\overline{T})$, and $\overline{\beta }=\beta (\overline{T})$. We now multiply Eq. (19) by $\overline{\psi }$ and Eq. (21) by $\psi$, subtract and integrate over all space:

22 $$\begin{aligned} \left( \beta ^{2}-\overline{\beta }^{2}\right) \int \overline{\psi }\psi \,dA = k^{2}\int \left( n^{2}-\overline{n}^{2}\right) \overline{\psi }\psi \,dA +\int {\left( \overline{\psi }\nabla _{t}^{2}\psi -\psi \nabla _{t}^{2} \overline{\psi } \right) }\,dA \end{aligned}$$

The last integral in Eq. (22) can be rewritten using Green’s second identity (Arfken et al. 2013):

23 $$\begin{aligned} \int \left( \overline{\psi }\nabla _t^2\psi -\psi \nabla _t^2\overline{\psi }\right) )\,dA&=\int {\nabla _{t}\cdot \left( \overline{\psi }\nabla _t\psi -\psi \nabla _t\overline{\psi }\right) \,dA}\nonumber \\&=\oint {\left( \overline{\psi }\nabla _{t}\psi -\psi \nabla _{t}\overline{\psi }\right) \cdot \hat{\textbf{n}}\,dl} \end{aligned}$$

In Eq. (23), *dl* denotes a line integral with outward normal $\hat{\textbf{n}}$ around the boundary of integration which is assumed to extend to infinity where $\psi$ and $\nabla _t\psi$ vanish. The line integral in Eq. (23) is then zero and Eq. (22) reduces to

24 $$\begin{aligned} \left( \beta ^2-\overline{\beta }^2\right) \int {\overline{\psi }\psi }\,dA = k^2\int {\left( n^2-\overline{n}^2\right) \overline{\psi }\psi \,dA} \end{aligned}$$

We now divide Eq. (24) by $\delta T$ and take the limit $\delta T \rightarrow 0$ to obtain

25 $$\begin{aligned} \frac{dn_{\text {eff}}^2}{dT}\int {\psi ^2dA=\int {\frac{dn^2}{dT}\psi ^2dA}} \end{aligned}$$

The modal normalisation is

26 $$\begin{aligned} \int \psi ^{2}\,dA=1 \end{aligned}$$

and in each cross sectional region $A_{i}$ the refractive index $n_{i}$ is a constant so that Eq. (25) can be expressed as:

27 $$\begin{aligned} \frac{dn_{\text {eff}}^2}{dT}=\sum _{i}{\frac{dn_i^2}{dT}\Gamma _i} \end{aligned}$$

where the power fraction in each region $A_{i}$ is given by

28 $$\begin{aligned} \Gamma _i=\int _{A_{i}}\psi ^2dA \end{aligned}$$

Equation (27) is the generalisation of Eq. (16) to an arbitrary weakly guiding waveguide and is an exact result. It is not the result of a perturbation analysis as $\psi$ is the exact wave function for the entire waveguide cross section and as the temperature changes the wave function can change significantly even though the scalar approximation remains valid. Equation (27) applies not only to fiber based waveguides, but to any weakly guiding structure such as a silica channel waveguide which we discuss in Sect. 6.4. For weak guidance $n_{1}\approx n_{2}$, and for the practical fiber devices which we discuss in the following sections, the temperature controlling regions have the same refractive index $n_{3}$. We can then simplify Eq. (27) to:

29 $$\begin{aligned} \frac{dn_{\text {eff}}^2}{dT}=\frac{dn_1^2}{dT}\left( \Gamma _1+\Gamma _2\right) +\frac{dn_3^2}{dT}\Gamma _3 \end{aligned}$$

where $\Gamma _3=\sum \Gamma _i$ and because $\Gamma _1+\Gamma _2+\sum \Gamma _i=1$ we obtain:

30 $$\begin{aligned} \frac{dn_{\text {eff}}^2}{dT}=\frac{dn_1^2}{dT}\left( 1-\Gamma _3\right) +\frac{dn_3^2}{dT}\Gamma _3 \end{aligned}$$

From Eq. (30) the condition for $dn_{\text {neff}}/dT=0$ is given by:

31 $$\begin{aligned} \Gamma _3=\dfrac{n_1\dfrac{dn_1}{dT}}{n_1\dfrac{dn_1}{dT}-n_3\dfrac{dn_3}{dT}} \end{aligned}$$

**Fig. 4:**
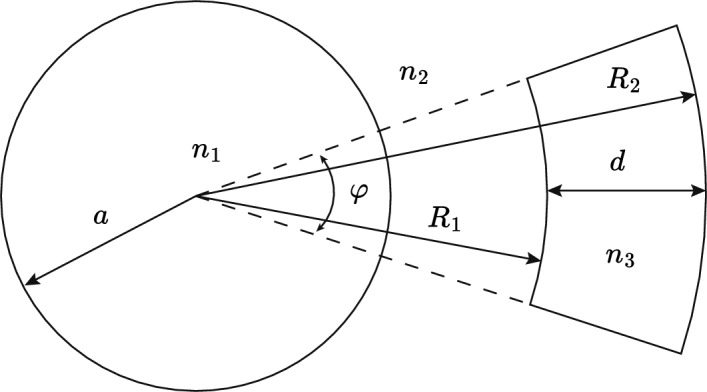
Geometry of a fiber with an annulus sector used for calculating modal fraction in the temperature controlling region $n_{3}$

Equation (31) has the same form as Eq. (17). It is interesting to note that Eq. (31) contains no details of the fiber construction, and that the value of $\Gamma _{3}$ required for $dn_{\text {eff}}/dT=0$ can be achieved by many different arrangements of the temperature controlling areas and waveguide geometry. This will become apparent in the examples discussed in the following sections. It is necessary to calculate $\Gamma _{3}$ at only one reference temperature, and is the only parameter that needs to be calculated in order to determine a temperature compensated design. Also, Eq. (31) can be rearranged so that if $\Gamma _{3}$ is known for a particular fiber design then the value of $dn_{3}/dT$ that is needed for a temperature compensated design can be determined.

## Thermal analysis of micro-channel fibers with matched cladding

**Fig. 5:**
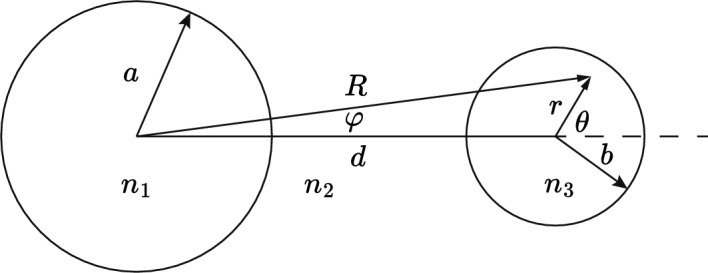
Geometry of a side hole fiber used for calculating modal fraction in the temperature controlling region $n_{3}$

In this section we consider the case of optical waveguides where the refractive index $n_{3}$ of the temperature controlling regions equals that of the cladding index $n_{2}$ at a reference temperature $T_{0}$. In this case there is an exact analytical solution for $\Gamma _{3}$ for many waveguides of practical interest and in particular for optical fibers with micro channels which are filled with a temperature controlling fluid or polymer. A wide range of suitable materials are available which can be matched to silica (Cargille; Chemoptics) and we discuss these in detail later in this paper. We consider two fiber geometries of practical interest: the first where the temperature controlling region is formed by the sector of an annulus, Fig. 4, and the second where the temperature control is produced by a side hole channel, Fig. 5.

### Matched cladding annulus sector fiber

The step index fiber analysed in Sect. 2 has limited application. By forming the temperature controlling region from a sector of an annulus surrounding the core a more practical geometry is achieved. This is shown in Fig. 4, where the inner and outer radii of the annulus are $R_{1}$ and $R_{2}$ respectively, and the annulus sector subtends an angle $\varphi$ at the center of the fiber core. Because $n_{3}=n_{2}$ at $T=T_{0}$, the mode function for $r>a$ is that of a step index fiber described by Eq. (3):

32 $$\begin{aligned} \psi =A\dfrac{J_{0}(u)}{K_{0}(w)}\cdot K_0{\left( \dfrac{wr}{a}\right) } {\text { for}}\ r>a \end{aligned}$$

The modal overlap $\Gamma _{3}$ with the annulus sector is given by:

33 $$\begin{aligned} \Gamma _{3} = \int _{R_{1}}^{R_{2}}\int _{0}^{\varphi }\psi ^{2}(r)\,rdr\,d\theta \end{aligned}$$

The integral in Eq. (33) is easily evaluated using the following result: (Gradshteĭn and Ryzhik 1980):

34 $$\begin{aligned} \int zK_{0}^{2}(z)\,dz=\frac{z^{2}}{2}\left[ K_{0}^{2}(z)-K_{1}^{2}(z)\right] \end{aligned}$$

Making use of Eq. (6), we find:

35 $$\begin{aligned} \Gamma _{3}= \frac{u^{2}\varphi }{2\pi V^{2}K_{1}^{2}(w)}\cdot \bigg \{\frac{R_{2}^{2}}{a^{2}}\Big [K_{0}^{2}\left( \frac{wR_{2}}{a}\right) - K_{1}^{2}\left( \frac{wR_{2}}{a}\right) \Big ]\bigg . \nonumber \\ -\frac{R_{1}^{2}}{a^{2}}\bigg .\Big [K_{0}^{2}\left( \frac{wR_{1}}{a}\right) - K_{1}^{2}\left( \frac{wR_{1}}{a}\right) \Big ] \bigg \} \end{aligned}$$

In the limit $\varphi =2\pi$, $R_{1}=a$, and $R_{2}=\infty$, Eq. (35) reduces to Eq. (8) as expected.

### Matched cladding side hole fiber

The geometry for a single side hole fiber is shown in Fig. 5 where two cylindrical coordinate systems $R,\phi$ and $r,\theta$ are shown. Because $n_{3}=n_{2}$ at $T=T_{0}$, the mode function for $R>a$ is again that of a step index fiber described by Eq. (3):

36 $$\begin{aligned} \psi =A\dfrac{J_{0}(u)}{K_{0}(w)}\cdot K_0{\left( \dfrac{wR}{a}\right) } {\text { for}}\ R>a \end{aligned}$$

where *R* is related to *r* by $R = \sqrt{r^{2}+d^{2}+2rd\cos \theta }$. The modal overlap $\Gamma _{3}$ with the side hole is then given by:

37 $$\begin{aligned} \Gamma _{3} = \int _{0}^{b}\int _{0}^{2\pi }\psi ^{2}(R)\,rdr\,d\theta \end{aligned}$$

To calculate the integral in Eq. (37) we use the addition theorem for Bessel functions so that Eq. (37) can be expressed in a single coordinate system (Watson 1995):

38 $$\begin{aligned} K_{0}\left( \frac{wR}{a}\right) = I_{0}\left( \frac{wr}{a}\right) K_{0}\left( \frac{wd}{a}\right) +2\sum _{p=1}^{\infty }(-1)^{p}I_{p}\left( \frac{wr}{a}\right) K_{p}\left( \frac{wd}{a}\right) \cos (p\theta ) \end{aligned}$$

Because the $\cos (p\theta )$ terms in Eq. (38) are orthogonal over $\theta = 0$ to $2\pi$, the integral of $K_{0}^{2}$ in Eq. (37) will only involve the sum of squares of the terms in Eq. (38). The integral over *r* in Eq. (37) can be calculated with the help of the following formula (Gradshteĭn and Ryzhik 1980):

39 $$\begin{aligned} \int zI_{p}^{2}(\lambda z)\,dz = \frac{z^{2}}{2}\left\{ I_{p}^{2}(\lambda z) - I_{p-1}(\lambda z)I_{p+1}(\lambda z) \right\} \end{aligned}$$

Combining Eq. (6) and Eqs. (36) to (39), and after some algebra, the following expression for $\Gamma _{3}$ is obtained:

40 $$\begin{aligned} \Gamma _{3}= & \frac{u^{2}b^{2}}{a^{2}V^{2}K_{1}^{2}(w)}\cdot \bigg \{K_{0}^{2}(\alpha )\Big [ I_{0}^{2}(\kappa )-I_{1}^{2}(\kappa ) \Big ]\bigg . \nonumber \\ & +\bigg .2\sum _{p=1}^{\infty }K_{p}^{2}(\alpha )\Big [I_{p}^{2}(\kappa )-I_{p-1}(\kappa ) I_{p+1}(\kappa ) \Big ] \bigg \} \end{aligned}$$

where $\alpha = wd/a$ and $\kappa = wb/a$. Although Eq. (40) involves an infinite sum over Bessel functions, the series converges rapidly and in practice only a few terms are needed.

**Fig. 6:**
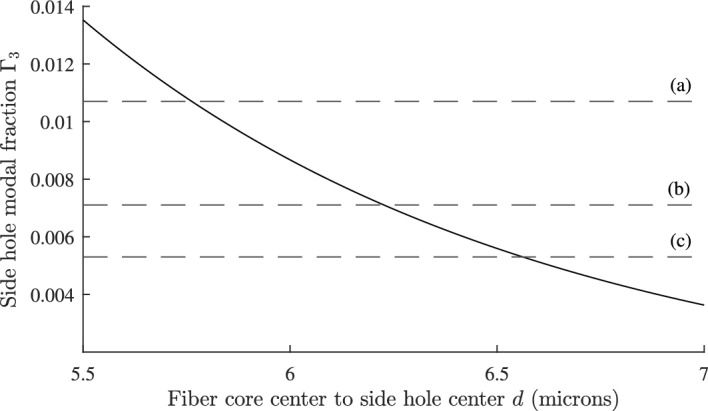
Plot of $\Gamma _{3}$ as a function of *d*, separation between core and side hole centers for the geometry in Fig. 5 with matched cladding $n_{3}=n_{2}$ at $T_{0}=25^{\circ } {\text {C}}$. The curve was calculated using the analytical result Eq. (40) for a fiber with a single side hole. The horizontal lines show where** a** $\Gamma _{3}=0.0107$,** b** $\Gamma _{3}=0.0071$, and** c** $\Gamma _3=0.0053$

### Design of temperature compensated optical fibers with matched cladding side hole fibers

The application of Eq. (35) and Eq. (40) to fiber design is similar and we restrict ourselves to the analysis of side hole fibers based on Eq. (40). Analogous results are obtained using Eq. (35). To illustrate how Eq. (40) can be used to design temperature compensated matched cladding fibers with temperature controlling side hole regions we consider the geometry of Fig. 5 with *a* = 4 µm, *b* = 1.5 µm, $n_{1}=1.451$, $n_{2}=1.445$, $dn_{1}/dT = dn_{2}/dT = 8.45\times 10^{-6}/^{\circ }\text {C}$ and $dn_{3}/dT = -389\times 10^{-6}/^{\circ }\text {C}$. For a matched cladding fiber, $n_{3}=n_{2}$ at the reference temperature $T_{0}=25^{\circ }\text {C}$. The refractive index and temperature dependence of $n_{3}$ is typical of siloxane and aliphatic / alicyclic hydrocarbon materials readily available from Cargille Laboratories, data sheet: Refractive-Index-Liquid-Series-AA-n$-$1.4540-at$-$589.3-nm-and-$25^{\circ }$C (Cargille).

**Fig. 7:**
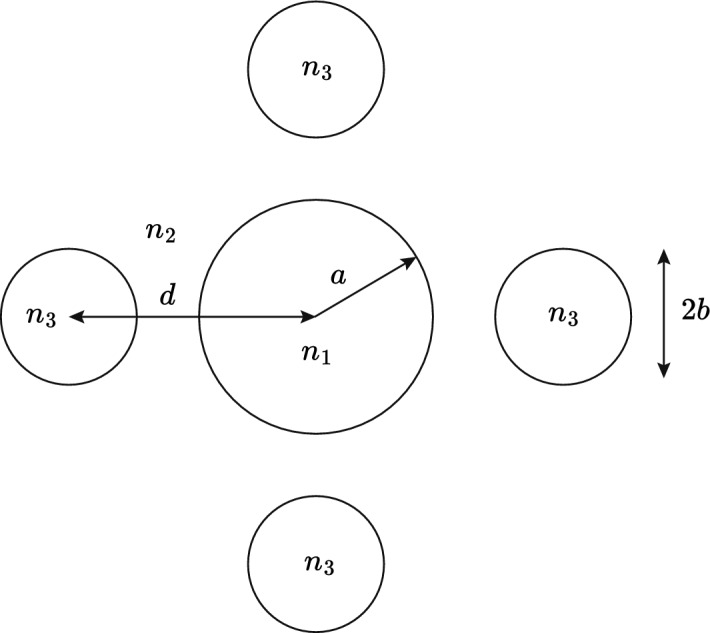
Geometry of four side hole fiber with side holes arranged symmetrically. The core and cladding have refractive indices $n_{1}$ and $n_{2}$ respectively, and the temperature controlling regions have the same refractive index *n*3

Figure 6 shows $\Gamma _{3}$, calculated using Eq. (40), as a function of *d* the fiber core center to side hole center. From Eq. (31) the value of $\Gamma _{3}$ for $dn_{\text {eff}}/dT=0$ is 0.0213. It can be seen in Fig. 6 that there is no value of *d* that achieves this for a single side hole. Because $n_{3}=n_{2}$ at $T_{0}$, the modal fraction $\Gamma _{3}$ is additive, so that a total $\Gamma _{3} = 0.0213$ can be achieved with two side holes, each with $\Gamma _{3}=0.0107$ for *d* = 5.76 µm and shown by the horizontal line (a). A second design is possible with three side holes each with $\Gamma _{3}=0.0071$, indicated by line (b) for *d* = 6.22 µm. A final design is possible with four side holes each with $\Gamma _{3}=0.0053$, and indicated by line (c) for *d* = 6.56 µm.

**Fig. 8:**
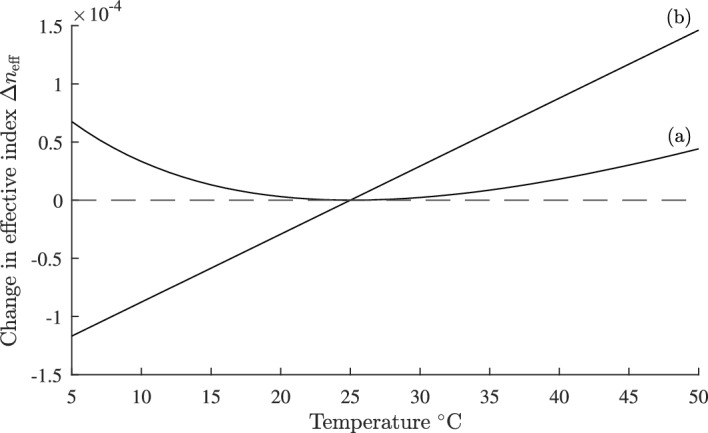
Plot of fractional change in effective index $\Delta n_{\text {eff}}$ against temperature for a matched cladding four side hole fiber. Curve** a** is for a temperature compensated design with parameters defined in Sect. 4.3, and curve** b** is for an uncompensated fiber where core and cladding both vary as $dn/dT = 8.45\times 10^{-6}/^{\circ }\text {C}$

We computed the temperature dependence of $\Delta n_{\text {eff}}$ for the four side hole geometry shown in Fig. 7 with the parameters given above, and with *d* = 6.56 µm, and a reference temperature $T_{0}=25^{\circ }\text {C}$. The results are shown in curve (a) in Fig. 8 and were computed using the finite difference module in FIMMWAVE (Photon Design). Identical results were achieved using the finite element module in FIMMWAVE. Also, shown for comparison in curve (b), is the temperature dependence of $\Delta n_{\text {eff}}$ when $dn/dT = 8.45\times 10^{-6}/^{\circ }\text {C}$ for all materials in the waveguide. Increasing the size or number of the sideholes will improve the temperature response. The single graph plotted in Fig. 6 allows all possible temperature compensated designs to be established for the given parameters without the need for repeated numerical calculations of the fiber temperature response.

## Thermal analysis of micro-channel fibers with unmatched cladding

**Fig. 9:**
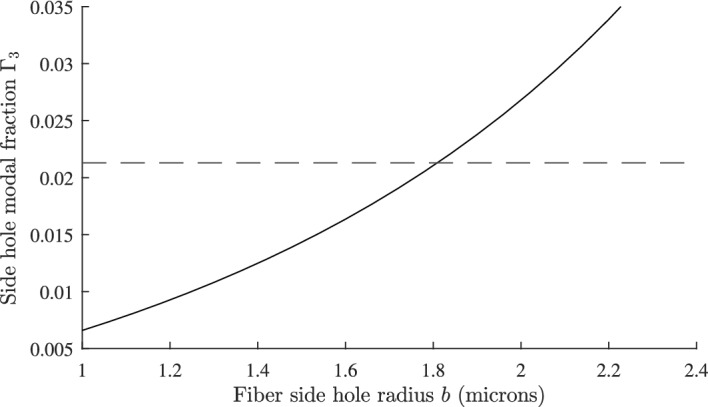
Plot of $\Gamma _{3}$ as a function of *b*, the side hole radius for the geometry in Fig. 7 with an unmatched cladding $n_{3} \ne n_{2}$ at $T_{0}=25^{\circ } {\textbf {C}}$. The separation *d* between the side hole center and fiber core center was set to the value *d* = 6.56 µm that was calculated in Subsection 4.3. The curve was calculated numerically for a fiber with four side holes. The horizontal lines show where $\Gamma _{3}=0.0213$

**Fig. 10:**
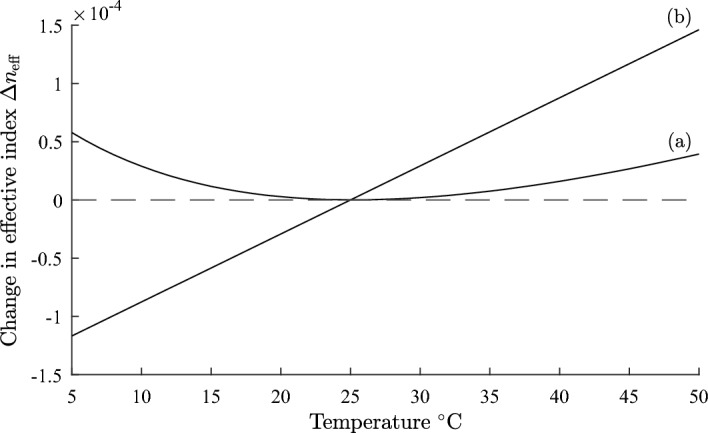
Plot of fractional change in effective index $\Delta n_{\text {eff}}$ against temperature for an unmatched cladding four side hole fiber. Curve** a** is for a temperature compensated design with parameters defined in Sect. 5, and curve** b** is for an uncompensated fiber where core and cladding both vary as $dn/dT = 8.45\times 10^{-6}/^{\circ }\text {C}$

When $n_{3} \ne n_{2}$ at $T_{0}$ it is no longer possible to calculate $\Gamma _{3}$ analytically for the geometry in Fig. 5. In order to then analyze the four side hole structure in Fig. 7 we must calculate $\Gamma _{3}$ numerically. This was computed using the finite difference module in FIMMWAVE (Photon Design) and the following parameters were chosen: *a* = 4 µm, $n_{1}=1.451$, $n_{2}=1.445$, and $n_{3}=1.44$, $dn_{1}/dT = dn_{2}/dT = 8.45\times 10^{-6}/^{\circ }\text {C}$ and $dn_{3}/dT = -391\times 10^{-6}/^{\circ }\text {C}$, corresponding to the Cargille refractive index data sheet: Refractive-Index-Liquid-Series-AA-n$-$1.4500-at$-$589.3-nm-and-$25^{\circ } \text {C}$ (Cargille). In order to make a direct comparison with the design in the previous section, we set the separation *d* between the side hole center and core center to *d* = 6.56 µm and calculate $\Gamma _{3}$ as a function of side hole radius *b*. From Eq. (31) we require $\Gamma _{3}=0.0213$. Figure 9 shows $\Gamma _{3}$ calculated as a function of *b* at $T_{0}=25^{\circ }\text {C}$. The horizontal line indicates where $\Gamma _{3}=0.0213$, corresponding to *b* = 1.81 µm. In Fig. 10 curve (a) we plot $\Delta n_{\text {eff}}$ as a function of temperature, and in curve (b) the corresponding result with no temperature compensation. Figure 10 shows an improvement over Fig. 8 of about $15\%$ at $T=5^{\circ }\text {C}$, reflecting the larger area occupied by the temperature compensating regions.

There have been a number of experimental demonstrations of fibers with microchannels filled with liquids. In one method fibers with microchannels along the length of the fiber have been made using the stack and draw method (Knight et al. 1996). By using capillary action the micro channels can be filled with suitable liquids over lengths of several cms. Using this approach, Huy et al. (2008) and (Naeem and Chung 2014) have demonstrated the temperature control of fiber Bragg gratings written into the core of the fiber. In Huy et al. (2008) fiber Bragg gratings with six and eighteen microchannels were demonstrated with the microchannels filled with liquids provided by Cargille Laboratories (Cargille). In Naeem and Chung (2014) the microchannels were in a photonic crystal fiber and were filled with methanol.

In a second approach the microchannels were formed by laser writing them in the fiber cladding, followed by etching (Song et al. 2025). Access points were provided at the surface of the fiber and the microchannels were liquid filled by capillary action over a length of 3 mm. In Song et al. (2025) fiber gratings with low temperature sensitivity were demonstrated.

We can estimate the device loss in the same way as in section 2.1. The quoted loss for the liquid in this section is $L=1.08 \text {dB/cm}$ (Cargille). For $\Gamma _{3}=0.0213$ the device loss is then estimated as $\Gamma _{3}L= 0.022 \text {dB/cm}$ (Adams 1981).

## Effects of thermal expansion

For a waveguide to be athermal the phase shift of the guided mode should be temperature compensated and this will be affected by thermal expansion. In this section we consider the effects of expansion in both transverse and longitudinal directions. Many optical waveguide devices and components are sensitive to the effects of thermal expansion. Typically, these make use of optical interference or phase change. For example, fiber Bragg gratings are widely used as sensors, including for strain and temperature, or as filters and demultiplexers in communication systems (Kersey et al. 1997). Arrayed waveguide grating demultiplexers based on planar silica-on-silicon technology (Leijtens et al. 2006) are sensitive to expansion of the waveguides, as well as thermal changes to the waveguide refractive indices. We consider the effects of thermal expansion in the transverse direction in Sect. 6.1 and in the longitudinal direction in Sect. 6.2 below.

### Transverse thermal expansion

The largest contribution to transverse thermal expansion will come from the waveguide core as the modal field takes its largest value there. For simplicity we consider a waveguide with a circular core with refractive index $n_{1}$, cladding with index $n_{2}$ and temperature controlling regions with refractive indices $n_{3}$ equal to $n_{2}$ at the reference temperature $T_{0}$. The core radius is *a*. This means that at temperature $T_{0}$ the waveguide mode is that of a step index fiber described by Eqs. (1) to (6). We have shown in Eqs. (25) and (26) that

41 $$\begin{aligned} \frac{dn_{\text {eff}}^2}{dT} =\int {\frac{dn^2}{dT}\psi ^2dA} \end{aligned}$$

We now express the radial dependence of $n^{2}$ at $T_{0}$ as a function of radius *r*:

42 $$\begin{aligned} n^{2}=n_{1}^{2}+\left( n_{2}^{2}-n_{1}^{2}\right) \cdot H\left( r-a\right) \end{aligned}$$

where *H* is unit the step function defined by

43 $$\begin{aligned} H(x) = {\left\{ \begin{array}{ll} 0& { \text { for }} x<0 ,\\ 1 & {\text { for }} x>0 \end{array}\right. } \end{aligned}$$

In Eq. (41) we can write $dn^{2}/dT$ as:

44 $$\begin{aligned} \frac{dn^{2}}{dT} = \frac{\partial n^{2}}{\partial T} + \frac{\partial n^{2}}{\partial r}\cdot \frac{\partial r}{\partial T} \end{aligned}$$

The contribution of the term ${\partial n^{2}}/{\partial T}$ in Eq. (44) has been given in Eq. (27), leaving the second term on the right hand side of Eq. (44). Using the properties of the step function this is given by

45 $$\begin{aligned} \frac{\partial n^{2}}{\partial r}\cdot \frac{\partial r}{\partial T} = (n_{2}^{2}-n_{1}^{2})\delta (r-a)\frac{\partial r}{\partial T} \end{aligned}$$

The integral in Eq. (41) is straightforward and involves $\psi (a)$, which can be found from Eq. (3). After some further algebra, and making use of Eq. (6), the condition for $dn_{\text {eff}}/dT$ = 0 is:

46 $$\begin{aligned} \Gamma _3=\dfrac{n_1\dfrac{dn_1}{dT}}{n_1\dfrac{dn_1}{dT}-n_3\dfrac{dn_3}{dT}} \times \left\{ 1+ \frac{\alpha (n_{1}^{2}-n_{2}^{2})}{n_{1}\dfrac{dn_{1}}{dT}}\cdot \frac{u^{2}}{V^{2}}\cdot \frac{K_{0}^{2}(w)}{K_{1}^{2}(w)}\right\} \end{aligned}$$

The coefficient of thermal expansion is $\alpha$, and *u*, *w*, and *V* are defined in Eqs. (1) and (2). The term in braces in Eq. (46) provides a correction to the expression for $\Gamma _{3}$ that was given in Eq. (31). To estimate a typical magnitude of the correction we choose parameters used previously: $\lambda$ = 1.55 µm, *a* = 4 µm, $n_{1}$ = 1.451, $n_{2}$ = 1.445, $dn_{1}/dT = 8.45\times 10^{-6}/^{\circ }\text {C}$, and $\alpha = 0.55\times 10^{-6}/^{\circ }\text {C}$ (Accuratus). From Eqs. (1) to (6) the following values can be determined: *V* = 2.1374, *u*/*V* = 0.7357, *w* = 1.4478, and $K_{0}(w)/K_{1}(w)$ = 0.7650. The correction term in Eq. (46) is then:

47 $$\begin{aligned} \frac{\alpha (n_{1}^{2}-n_{2}^{2})}{n_{1}\dfrac{dn_{1}}{dT}}\cdot \frac{u^{2}}{V^{2}}\cdot \frac{K_{0}^{2}(w)}{K_{1}^{2}(w)} = 0.00025 \end{aligned}$$

For most applications the correction in Eq. (47) can be neglected. An analysis is possible for a general waveguiding structure, but it is more complex and leads to a similar result.

### Longitudinal thermal expansion

The effects of longitudinal thermal expansion can be expressed in terms of the phase change $\phi$ along a length *L* of a waveguide:

48 $$\begin{aligned} \phi = n_{\text {eff}}kL \end{aligned}$$

where *k* is the free space wave number. Differentiating Eq. (48) and rearranging we obtain:

49 $$\begin{aligned} \frac{1}{\phi }\frac{d\phi }{dT}=\frac{1}{2n_{\text {eff}}^{2}}\frac{dn_{\text {eff}}^{2}}{dT}+\alpha \end{aligned}$$

Combining Eqs. (49) and (30) the condition for $d\phi /dT=0$ is:

50 $$\begin{aligned} \Gamma _3=\dfrac{n_1\dfrac{dn_1}{dT}}{n_1\dfrac{dn_1}{dT}-n_3\dfrac{dn_3}{dT}}\cdot \left\{ 1+\frac{\alpha n_{\text {eff}}^{2}}{n_{1}\dfrac{dn_{1}}{dT}}\right\} \end{aligned}$$

Equation (50) is exact within the weakly guiding approximation, and does not rely on perturbation methods. Using the waveguide parameters in the previous section, and noting that $n_{1}\ge n_{\text {eff}}\ge n_{2}$ and $n_{1}\approx n_{2}$ the correction to $\Gamma _{3}$ due to longitudinal expansion can be accurately approximated by

51 $$\begin{aligned} \frac{\alpha n_{\text {eff}}^{2}}{n_{1}\dfrac{dn_{1}}{dT}}\approx \alpha n_{1}\cdot \frac{1}{\dfrac{dn_{1}}{dT}} = 0.094 \end{aligned}$$

With this approximation, Eq. (50) is independent of the waveguide geometry and can be written as:

52 $$\begin{aligned} \Gamma _3=\dfrac{n_1\dfrac{dn_1}{dT}}{n_1\dfrac{dn_1}{dT}-n_3\dfrac{dn_3}{dT}}\cdot \left\{ 1+\alpha n_{1}\cdot \frac{1}{\dfrac{dn_{1}}{dT}}\right\} \end{aligned}$$

Equation (52) represents about a $10\%$ increase in $\Gamma _{3}$ compared to no thermal expansion, and if not compensated will result in a shift of the center wavelength of a fiber Bragg grating or arrayed waveguide grating. We discuss this in the following sections.

**Fig. 11:**
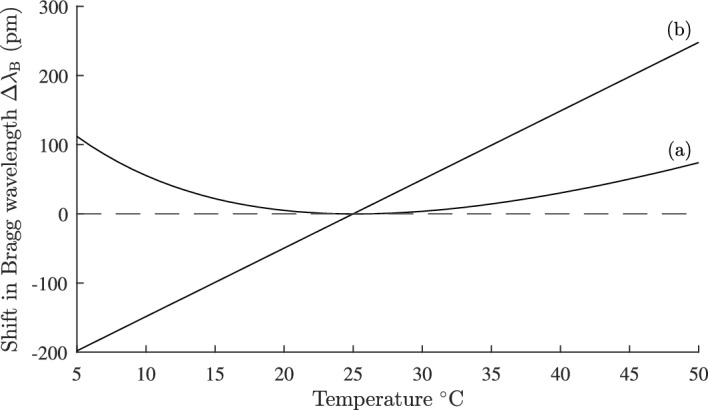
Shift in picometers of the Bragg wavelength with temperature for a fiber Bragg grating with the fiber geometry in Fig. 7. Curve** a** is for an athermal design, and curve** b** for an uncompensated design. Parameters used are given in Sects. 6.1 and 6.3

### Application to fiber Bragg gratings

For a fiber grating with period $\Lambda$, the Bragg wavelength $\lambda _{\text {B}}$ is given by Kersey et al. (1997):

53 $$\begin{aligned} \lambda _{\text {B}}=2n_{\text {eff}}\Lambda \end{aligned}$$

The condition for $d\lambda _{\text {B}}/dT$ = 0 is given by Eq. (52) at a reference temperature $T_{0}$. At temperature *T* the Bragg wavelength is $\lambda _{\text {B}}(T)$, and at temperature $T_{0}$ it is $\lambda _{\text {B}}(T_{0})$. The shift $\Delta \lambda _{\text {B}}=\lambda _{\text {B}}(T)-\lambda _{\text {B}}(T_{0})$ of the Bragg wavelength with temperature is given by:

54 $$\begin{aligned} \Delta \lambda _{\text {B}}=\lambda _{\text {B}}(T_{0})(\Delta n_{\text {eff}}+\alpha (T-T_{0})) \end{aligned}$$

where $\Delta n_{\text {eff}}$ was defined in Eq. (18). We consider the geometry of Fig. (7), with $n_{3}=n_{2}$ and $dn_{3}/dT = -389\times 10^{-6}/^{\circ }\text {C}$ at $T_{0}=40^{\circ }\text {C}$, *b* = 1.5 µm and the other parameters as given in Sect. 6.1. From Eq. (52) the required value of $\Gamma _{3}$ = 0.0233. Calculating Eq. (40) and computing a similar graph to Fig. (6), the required separation *d* between core and side hole centers is found to be *d* = 6.45 µm. Using Eq. (54) the change in $\Delta \lambda _{\text {B}}$ with temperature is shown in Fig. 11 curve (a). Also shown for comparison in curve (b) is the change in Bragg wavelength if there is no temperature compensation. The athermal design reduces the maximum shift in $\Delta \lambda _{\text {B}}$ by about 100 pm. Equation (50) allows many other designs to be quickly found without the need to perform multiple finite element simulations of the full temperature response. Equally important, Eq. (50) allows the rapid elimination of unsuccessful designs. It is interesting to note $\Gamma _{3}$ is largest at low temperatures, and falls with increasing temperature, which implies that the grating reflectivity will be slightly reduced at low temperatures.

**Fig. 12:**
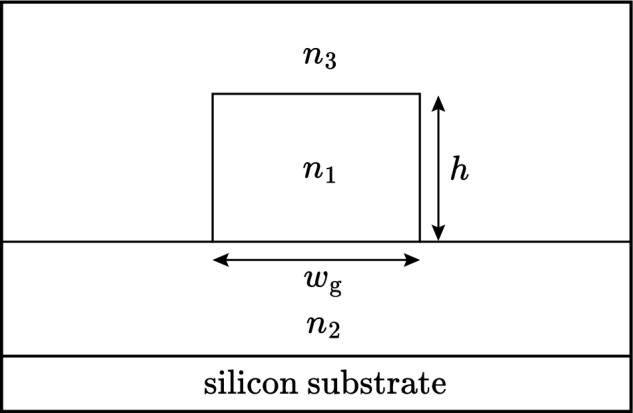
Cross section of a silica on silicon waveguide. The waveguide materials $n_{1}, n_{2} \text { and } n_{3}$ are normally silica or germania doped silica, grown on a silicon wafer. An athermal waveguide design results from replacing $n_{3}$ with a suitable material with a negative thermal coefficient for the refractive index

**Fig. 13:**
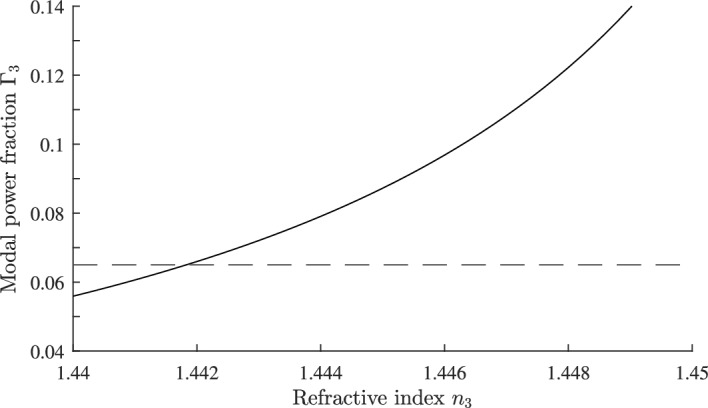
Plot of $\Gamma _{3}$ as a function of $n_{3}$ with $dn_{3}/dT = -180\times 10^{-6}/^{\circ }\text {C}$ for a silica on silicon waveguide with $n_{1}=1.4574$, $n_{2}=1.453$, $dn_{1}/dT = dn_{2}/dT= 8.45\times 10^{-6}/^{\circ }\text {C}$ and $w_g=h$ = 8 µm. The coefficient of thermal expansion of the silicon substrate is $\alpha _{\text {sub}} = 2.63\times 10^{-6}/^{\circ }\text {C}$. The horizontal line corresponds to $\Gamma _{3}=0.065$

### Application to silica arrayed waveguide gratings

The arrayed waveguide grating (AWG) is widely used in optical fiber communication systems, with applications including demultiplexing or multiplexing in wavelength division multiplexing (WDM) networks, as well as in other routing devices. A detailed review of AWG technology and design can be found in Leijtens et al. (2006) and (Okamoto 2000). Focusing in an AWG is achieved at a central wavelength $\lambda _{\text {c}}$:

55 $$\begin{aligned} \lambda _{\text {c}}=\frac{n_{\text {eff}}\Delta L}{m} \end{aligned}$$

where $\Delta L$ is the length difference between adjacent array waveguides, $n_{\text {eff}}$ is the waveguide effective index and *m* is the diffraction order (Leijtens et al. 2006). The central wavelength, together with the AWG geometry, determines the channel spacing, and free spectral range. A widely used waveguide technology for fabricating AWGs is silica-on-insulator (SOI) (Himeno et al. 1998; Ou 2003). Figure 12 shows the cross section of a typical SOI waveguide grown on a silicon substrate. Normally, the materials $n_{1}$, $n_{2}$, and $n_{3}$ are silica or germania doped silica, and because of the temperature dependence of $n_{\text {eff}}$, and thermal expansion of $\Delta L$, the central wavelength $\lambda _{\text {c}}$ varies with temperature so that:

56 $$\begin{aligned} \frac{1}{\lambda _{\text {c}}}\frac{d\lambda _{ \text {c}}}{dT}= \frac{1}{2n_{\text {eff}}^{2}}\frac{dn_{\text {eff}}^{2}}{dT} + \alpha _{\text {sub}} \end{aligned}$$

The coefficient of thermal expansion of the silicon substrate is $\alpha _{\text {sub}}$. In this section we demonstrate how the results derived in this paper can be applied to the design of an athermal AWG by using a polymer for the superstrate material $n_{3}$, Inoue et al. (1997); Li et al. (2007); Wang et al. (2009). Comparing Eqs. (49) and (56) the condition for $d\lambda _\text {c}/dT=0$ is given by Eq. (52) with $\alpha$ replaced by $\alpha _{\text {sub}}$. Referring to Fig. 12 we choose $n_{1}=1.4574$, $n_{2}=1.453$, $dn_{1}/dT = dn_{2}/dT= 8.45\times 10^{-6}/^{\circ }\text {C}$ and $w_\text {g}=h$ = 8 µm. This corresponds to a core substrate index difference of 0.3% and *t* and *h* chosen so that the modal field matches that of a SMF28 communications fiber (Leijtens et al. 2006). The superstrate $n_{3}$ is a polymer such as ZPU 13-RI with $dn_{3}/dT = -180\times 10^{-6}/^{\circ }\text {C}$ and is available with $n_{3}$ in the range 1.43$-$1.46 from Chemoptics, data sheet: Exguide LFR/ZPU12, 13-RI series (Chemoptics). The coefficient of thermal expansion for silicon is $\alpha _{\text {sub}} = 2.63\times 10^{-6}/^{\circ }\text {C}$ (Li et al. 2007; Keil et al. 2001). The reference temperature is set at $T_{0}=40^{\circ }\text {C}$ and from Eq. (52) the value of $\Gamma _{3}$ so that $d\lambda _\text {c}/dT=0$ is calculated to be $\Gamma _{3}=0.065$. Figure 13 shows part of a plot of $\Gamma _{3}$ computed as a function of $n_{3}$, from which we find $n_{3}=1.442$ for $\Gamma _{3}=0.065$. The shift $\Delta \lambda _{\text {c}}=\lambda _{\text {c}}(T)-\lambda _{\text {c}}(T_{0})$ of the center wavelength is:

57 $$\begin{aligned} \Delta \lambda _{\text {c}}=\lambda _{\text {c}}(T_{0})(\Delta n_{\text {eff}}+\alpha _{\text {sub}}(T-T_{0})) \end{aligned}$$

**Fig. 14:**
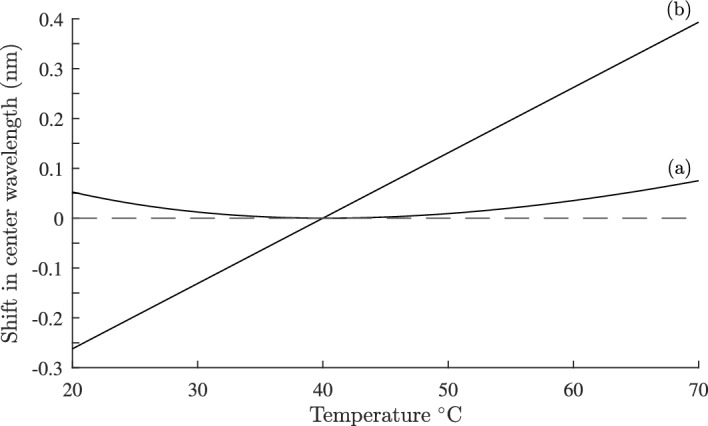
Shift in nanometers of the center wavelength with temperature for an arrayed waveguide grating based on silica on insulator technology with the parameters described in the text and Fig. 13. Curve** a** is the athermal design, and curve** b** is the response for an uncompensated design

This is plotted in Fig. 14, curve (a) as a function of temperature over the range $T=20\text { to }70 ^{\circ }\text {C}$. With temperature compensation the shift in $\lambda _{\text {c}}$ is about 0.05 nm. For comparison in curve (b), there is a shift of more than 0.6 nm in $\lambda _{\text {c}}$ across the same temperature range when there is no temperature compensation. A similar result, computed by a different approach, was reported in Inoue et al. (1997); Li et al. (2007).

The loss of the polymer ZPU 13-RI is quoted as $L = 0.31\text {dB/cm}$ (Chemoptics), and with $\Gamma _{3}=0.065$ corresonds to a device loss of $\Gamma _{3}L=0.02\text {dB/cm}$.

## Analysis of strongly guiding waveguides with vector modes

The analysis presented so far applies to weakly guiding waveguides where there is a small difference between refractive indices. The modal fields are accurately described by a scalar wave function and wave equation. For strongly guiding waveguides where the refractive index differences are large, such as silicon-on-insulator (Jalali et al. 1998), the modes must be described by fully vector electric and magnetic fields and Eq. (27) will no longer apply. In this section we derive an expression for $dn_{\text {eff}}/dT$ that is valid for a fully vector theory. It is straightforward to extend the analysis that follows to a general anisotropic waveguide.

We assume a time dependence $\text {exp}(i\omega t)$ for the fields and Maxwell’s equations are given by:

58 $$\begin{aligned} \nabla \times \textbf{E}&=-i\omega \mu _{0}\textbf{H} \end{aligned}$$

59 $$\begin{aligned} \nabla \times \textbf{H}&=i\omega \varepsilon _{0}n^{2}\textbf{E} \end{aligned}$$

The electric and magnetic fields at temperature *T* are $\textbf{E}$ and $\textbf{H}$, and at temperature $\overline{T}=T+\delta T$ they are $\overline{\textbf{E}}$ and $\overline{\textbf{H}}$. We introduce the vector $\textbf{F}$ defined by Snyder and Love (1984):

60 $$\begin{aligned} \textbf{F}=\textbf{E}\times \overline{\textbf{H}}^{\,*} + \mathbf {\overline{E}}^{\,*}\times \textbf{H} \end{aligned}$$

Using the two-dimensional form of the divergence theorem, the following relation holds:

61 $$\begin{aligned} \int _{A} \nabla \cdot \textbf{F}\,dA=\frac{\partial }{\partial z}\int _{A}\textbf{F}\cdot \hat{\textbf{z}}\,dA+\oint \textbf{F}\cdot \hat{\textbf{n}}\,dl \end{aligned}$$

where *A* is the waveguide cross section, $\hat{\textbf{z}}$ is the unit vector along the waveguide, and the line integral is around the boundary of *A* with $\hat{\textbf{n}}$ the outward normal unit vector on *l*. If we let the cross section *A* be the infinite cross section $A_{\infty }$, and assume that the fields vanish on the boundary, then the line integral vanishes and Eq. (61) becomes

62 $$\begin{aligned} \int _{A_{\infty }} \nabla \cdot \textbf{F}\,dA=\frac{\partial }{\partial z}\int _{A_{\infty }}\textbf{F}\cdot \hat{\textbf{z}}\,dA \end{aligned}$$

The electric and magnetic fields can be written in terms of the waveguide modal fields $\textbf{e}$ and $\textbf{h}$ at temperature *T* as:

63 $$\begin{aligned} \textbf{E}=\textbf{e}\,\text {exp}(-i\beta z) \end{aligned}$$

64 $$\begin{aligned} \textbf{H}=\textbf{h}\,\text {exp}(-i\beta z) \end{aligned}$$

and at temperature $\overline{T}=T+\delta T$ as:

65 $$\begin{aligned} \overline{\textbf{E}}=\overline{\textbf{e}}\,\text {exp}(-i\overline{\beta } z) \end{aligned}$$

66 $$\begin{aligned} \overline{\textbf{H}}=\overline{\textbf{h}}\,\text {exp}(-i\overline{\beta } z) \end{aligned}$$

where $\overline{\beta }=\beta (T+\delta T)$. The modal fields $\textbf{e}$ and $\textbf{h}$ are normalised so that:

67 $$\begin{aligned} \int _{A_{\infty }}\textbf{e}\times \textbf{h}^{*}\mathbf {\cdot }\hat{\textbf{z}}\,dA =\int _{A_{\infty }}\textbf{e}^{*}\times \textbf{h}\mathbf {\cdot }\hat{\textbf{z}}\,dA = 1 \end{aligned}$$

We evaluate the integrands on each side of Eq. (62). Taking the divergence of Eq. (60) we have:

68 $$\begin{aligned} \nabla \cdot \textbf{F}=\overline{\textbf{H}}^{\,*}\cdot \nabla \times \textbf{E}-\textbf{E}\cdot \nabla \times \overline{\textbf{H}}^{\,*} +\textbf{H}\cdot \nabla \times \overline{\textbf{E}}^{\,*}-\overline{\textbf{E}}^{\,*}\cdot \nabla \times \textbf{H} \end{aligned}$$

Using Eqs. (58 ), (59) and Eqs. (63) to (66) we can simplify Eq. (62):

69 $$\begin{aligned} \nabla \cdot \textbf{F}=i\omega \epsilon _{0}\left( n^{2}-\overline{n}^{2}\right) \textbf{e}\cdot \mathbf {\overline{e}}^{\,*}\text {exp}\left( i(\overline{\beta }-\beta ) z\right) \end{aligned}$$

where $\overline{n}=n(T+\delta T)$. Using Eqs. (63) to (66) we find:

70 $$\begin{aligned} \frac{\partial \textbf{F}}{dz}=\left( i(\overline{\beta }-\beta )\right) (\textbf{e}\times \overline{\textbf{h}}^{\,*}+\overline{\textbf{e}}^{\,*}\times \textbf{h})\text {exp}\left( i(\overline{\beta }-\beta ) z\right) \end{aligned}$$

Combining Eqs. (62), (69) and (70), together with the normalisation condition Eq. (67), and taking the limit $\delta T\rightarrow 0$ so that $\overline{\textbf{e}}\rightarrow \textbf{e}$ and $\overline{\textbf{h}}\rightarrow \textbf{h}$ we find:

71 $$\begin{aligned} \frac{dn_\text {eff}}{dT}=\frac{1}{2}\sqrt{\frac{\epsilon _{0}}{\mu _{0}}}\int _{A_{\infty }}\frac{dn^{2}}{dT}\mid \textbf{e}\mid ^{2}\,dA \end{aligned}$$

If $n=n_{i}$ in each region $A_{i}$ of the waveguide, we can rewrite Eq. (71) as follows:

72 $$\begin{aligned} \frac{dn_\text {eff}}{dT}=\frac{1}{2}\sqrt{\frac{\epsilon _{0}}{\mu _{0}}}\sum _{i}\frac{dn_{i}^{2}}{dT}\int _{A_{i}}\mid \textbf{e}\mid ^{2}\,dA \end{aligned}$$

The extension of this analysis to anisotropic fibers is straightforward and in place of Eq. (71) we find:

73 $$\begin{aligned} \frac{dn_\text {eff}}{dT}=\frac{1}{2}\sqrt{\frac{\epsilon _{0}}{\mu _{0}}}\int _{A_{\infty }}e_{k}\frac{dn^{2}_{kl}}{dT}e^{*}_{l}\,dA \end{aligned}$$

The refractive index tensor $n^{2}_{kl}$ is related to the dielectric tensor $\epsilon _{kl}$ by $n^{2}_{kl}=\epsilon _{kl}$, and summation over the repeated indices *k* and *l* is implied in Eq. (73).

Equation (72) is exact and should be compared to the weakly guiding result Eq. (27). An important difference is that in general, the integral in Eq. (72) is not the fraction of modal power in the cross section $A_{i}$, which means that Eq. (27) should not be applied to a strongly guiding waveguide such as silicon on insulator (Jalali et al. 1998). An exception is the TE mode on a planar optical waveguide when it is straightforward to show that Eq. (72) is the same as Eq. (27).

We shall now show transverse thermal expansion of a strongly guiding waveguide is no longer negligible, as was demonstrated in Sect. 6.1 for the case of weak guidance. If we repeat the analysis of Sect. 6.1 and use Eqs. (41) to (45) in Eq. (71), we can estimate the contribution of transverse thermal expansion of the waveguide core to $dn_{\text {eff}}/dT$ from Eq. (71) as:

74 $$\begin{aligned} \pi a^{2}\sqrt{\frac{\epsilon _{0}}{\mu _{0}}}\alpha (n_{1}^{2}-n_{2}^{2})\mid \textbf{e}(a)\mid ^{2} \end{aligned}$$

For a silicon waveguide, the coefficient of thermal expansion $\alpha = 2.63\times 10^{-6}/^{\circ }\text {C}$, and $(n_{1}^{2}-n_{2}^{2})\approx 10$, compared to the values for silica used in Sect. 6.1 of $\alpha = 0.55\times 10^{-6}/^{\circ }\text {C}$ and $(n_{1}^{2}-n_{2}^{2})=0.017$. This is a ratio of about 2780 which implies that transverse thermal expansion effects are expected to be significant for strongly guiding structures.

Finally, we show that Eq. (72) reduces to Eq. (27) in the weak guidance limit. In a weakly guiding waveguide $\textbf{e}$ and $\textbf{h}$ are orthogonal and transverse to the z direction, so that they can be written as:

75 $$\begin{aligned} \textbf{e}&=\hat{\textbf{x}}\,e_{x}\,\text {exp}(-i\beta z) \end{aligned}$$

76 $$\begin{aligned} \textbf{h}&=\hat{\textbf{y}}\,h_{y}\,\text {exp}(-i\beta z) \end{aligned}$$

where $\hat{\textbf{x}}$ and $\hat{\textbf{y}}$ are unit vectors along the x and y axes. The normalization condition Eq. (67) is then:

77 $$\begin{aligned} \int _{A_{\infty }}e_{x}h_{y}^{*}\,dA=1 \end{aligned}$$

Taking the y-component of Eq. (58) we find that $e_{x}$ can be expressed in terms of $h_{y}$ as:

78 $$\begin{aligned} e_{x}=\frac{1}{n_{\text {eff}}}\sqrt{\frac{\mu _{0}}{\epsilon _{0}}}\,h_{y} \end{aligned}$$

Then we can write $\mid \textbf{e}\mid ^{2}$ as:

79 $$\begin{aligned} \mid \textbf{e}\mid ^{2}\,=\frac{1}{n_{\text {eff}}}\sqrt{\frac{\mu _{0}}{\epsilon _{0}}}e_{x}h_{y}^{*} \end{aligned}$$

Equation (72) then becomes

80 $$\begin{aligned} \frac{dn_\text {eff}}{dT}=\frac{1}{2n_{\text {eff}}}\sum _{i}\frac{dn_{i}^{2}}{dT}\int _{A_{i}}e_{x}h_{y}^{*}\,dA \end{aligned}$$

Because of the normalization condition Eq. (77), the integral in Eq. (80) is $\Gamma _{i}$, the fraction of modal power in the cross section $A_{i}$, which finally gives us

81 $$\begin{aligned} \frac{dn_\text {eff}^{2}}{dT}=\sum _{i}\frac{dn_{i}^{2}}{dT}\Gamma _{i} \end{aligned}$$

This is the same as the scalar result Eq. (27).

## Conclusion

In this paper we have presented an exact analysis of the temperature dependence of optical waveguides with thermo-optic controlling regions, either as micro channels around a fiber core, or as cladding regions in a planar waveguide structure. We have shown that the condition for zero rate of change with temperature of the waveguide effective index is determined by a simple but exact equation that relates the temperature variation of the effective index to the fractions of modal field in the different waveguide regions. This condition is independent of the waveguide structure and provides a unified approach for determining temperature compensated designs, or waveguide structures for which no such design is possible.

Our analysis requires the computation of a single parameter at one temperature: the modal power fraction in the temperature compensating regions. Some waveguides of practical interest have exact solutions, as described in Sects. 2 and 4, and in these cases a complete analytical solution is possible for a temperature compensated waveguide design. For those waveguides for which no analytical solution exists we have calculated the modal power fraction using finite difference or finite element methods, such as FIMMWAVE (Photon Design). Again, only one parameter at a single temperature needs to be calculated. This can be contrasted with analyses using finite element methods where the waveguide behavior must be repeatedly calculated over a range of temperatures (Gylfason et al. 2012; Man et al. 2019; Mothe et al. 2008; Wang et al. 2009).

We have shown how the effects of thermal expansion can be included in order to produce athermal designs. We applied our analysis to a variety of optical waveguiding structures and devices but without attempting to produce optimised designs.

We extended the analysis to strongly guiding waveguides where the modal field must be described by a full vector equation. The condition for $dn_{\text {eff}}/dT=0$ can no longer be written in terms of the fractions of modal power in each refractive index region, and Eq. (27) should not be used for strongly guiding waveguides. The effects of transverse thermal expansion can no longer be ignored. Finally, we demonstrated that the vector result reduces to the scalar result Eq. (27) in the weak guidance limit.

## Data Availability

No datasets were generated or analysed during the current study.
